# Evaluation of Gastroprotective Activity of the Methanolic Extract of *Justicia pectoralis* Jacq. (Acanthaceae)

**DOI:** 10.3390/nu16101430

**Published:** 2024-05-09

**Authors:** Ismael Aureliano Rosa Júnior, Dionys de Souza Almeida, Hamilton Barbosa Napolitano, Josana de Castro Peixoto, Lucimar Pinheiro Rosseto, Emerith Mayra Hungria Pinto, Lucas Danilo Dias, James Oluwagbamigbe Fajemiroye, Elson Alves Costa, Rodolfo P. Vieira, José Luis Rodrigues Martins

**Affiliations:** 1 Postgraduate Program in Pharmaceutical Sciences, Pharmacology and Therapeutics, Evangelical University of Goiás—Unievangélica, University Avenue Km 3,5, Anápolis 75083-515, GO, Brazil; ismael@ictq.com.br (I.A.R.J.); hamilton.napolitano@docente.unievangelica.edu.br (H.B.N.); josana.peixoto@gmail.com (J.d.C.P.); lucimar.rosseto@unievangelica.edu.br (L.P.R.); emerith0706@hotmail.com (E.M.H.P.); lucasdanillodias@gmail.com (L.D.D.); jamesfajemiroye@ufg.br (J.O.F.); rodrelena@yahoo.com.br (R.P.V.); 2 Instituto de Ciência, Tecnologia e Qualidade—ICTQ, Anápolis 75023-085, GO, Brazil; 3 Institute of Biological Sciences, Department of Pharmacology, Federal University of Goiás, Campus Samambaia, Campus Street, Goiânia 74001-97, GO, Brazil; souzadionys@discente.ufg.br (D.d.S.A.); xico@ufg.br (E.A.C.); 4 Campus Central, State University of Goiás, Anápolis 75132-400, GO, Brazil; 5 Postgraduate Program in Human Movement and Rehabilitation, Evangelical University of Goiás—Unievangélica, University Avenue Km 3,5, Anápolis 75083-515, GO, Brazil

**Keywords:** *Justicia pectoralis* Jacq., Acanthacea, gastroprotective activity, gastric ulcer, methanolic extract

## Abstract

Introduction: *Justicia pectoralis* Jacq. is traditionally applied in folk medicine in Brazil and in several Latin American countries. The leaves are used in tea form, especially in the treatment of respiratory disorders, acting as an expectorant. It also has activity in gastrointestinal disorders, and it is anti-inflammatory, antioxidant, sedative, and estrogenic, among others. Aims: To investigate the gastroprotective activity of the methanol extract of the leaves of *Justicia pectoralis* Jacq. (MEJP) in different experimental models of gastric ulcers. Materials and methods: The adult leaves of *Justicia pectoralis* Jacq. were collected and cultivated in beds, with an approximate spacing of 40 × 40 cm, organic fertilization, irrigation with potable water and without shelter from light. The MEJP was prepared from the dried and pulverized leaves and concentrated under reduced pressure in a rotary evaporator. For the experimental model of gastric ulcer, Swiss male albino mice were used. The inputs used in the experiment were MEJP at three different concentrations (250, 500 and 1000 mg/kg p.o.), cimetidine (50 mg/kg p.o.), indomethacin (50 mg/kg s.c.) and vehicle (10 mL/kg p.o.). Results: MEJP (250, 500 and 1000 mg/kg p.o.) demonstrated gastroprotective activity, with levels of protection of 45.65%, 44.80% and 40.22%, respectively, compared to the control (vehicle). Compared with cimetidine (48.29%), MEJP showed similar gastroprotective activity. Conclusions: This study demonstrated the gastroprotective activity of MEJP and contributes to validate the traditional use the species for gastric disorders and provides a pharmacological basis for its clinical potential.

## 1. Introduction

Peptic ulcers have been a major cause of morbidity and mortality for centuries, causing life-threatening complications such as perforation and bleeding of the gastric mucosa [1,2]. This gastrointestinal disorder affects annually about 14.5 million people and its physiopathology is based on imbalance between the defense mechanisms of the gastric mucosa, including mucus synthesis, bicarbonate, prostaglandins (PGs), maintenance of blood flow, and offensive mechanisms such as secretion of gastric acid and pepsin [3,4].

Conventional treatments usually improve gastric ulcer recovery, such as histamine H2 receptor antagonists (cimetidine, famotidine and ranitidine) and proton pump inhibitors (IBPs) (lansoprazole, esomeprazole and omeprazole); however, both drugs are closely linked to ulcer recurrence, as these treatments are insufficient for the complete cure of the disease [5]. Furthermore, the prolonged use of these drugs can lead to a series of side effects such as hepatotoxicity, chronic kidney disease, vitamin B12 deficiency, gynecomastia, impotence and increased risk of fracture [6,7].

In this regard, new therapeutic proposals have been tested against peptic ulcers such as plant extracts and their isolated constituents [3,7]. Among them, *Justicia pectoralis* Jacq., which belongs to the *Acanthaceae* family, is a perennial herbaceous plant, popularly known as “chambá” or “anador” being used in traditional medicine in several countries in South and Central America [8,9,10,11,12,13]. In Central American countries, the plant is popularly used as tea, has expectorant and anxiolytic activity, and reduces several problems related to the prostate [10].

Several authors have highlighted the different types of pharmacological properties of the species, ranging from sedative, antiepileptic, estrogenic, including bactericidal and antiviral, antinociceptive, anti-inflammatory, and antioxidant [11,12,14,15,16,17], as well as having antiplatelet activity and an effect on gastrointestinal disorders, with it being considered a candidate for the treatment of peptic ulcers [10,13,15,16,17,18].

On this subject, this paper aims to investigate the gastroprotective activity of a methanolic extract of the leaves of *Justicia pectoralis* Jacq. (MEJP) using different experimental models of induced gastric ulcers.

## 2. Materials and Methods

### 2.1. Collection of Botanical Material

The leaves of *Justicia pectoralis* Jacq. were collected in the Vegetation House at Evangelical University of Goiás—UniEVANGÉLICA, Anápolis, Goiás (16°34′50″ S and 48°92′88″ W), in February 2017. Taxonomic identification was performed by the experienced Prof. Dr. Joana de Castro Peixoto. The cultivation was carried out in beds, with approximate spacing of 40 × 40 cm, organic fertilization, irrigation with potable water and without shelter from light. The plant matrices were also collected in the Vegetation House of the University of Brasília (UnB). Exsiccates of the leaves of *Justicia pectoralis* Jacq. were deposited in the herbarium of the State University of Goiás (UEG), Campus Anápolis, Goiás, under number 1234. Fresh adult leaves that did not show signs of necrosis, chlorosis or fungal contamination were collected. For greater sample uniformity, the same batch of samples was used and collections were always performed at 10 a.m.

### 2.2. Extract Preparation

The leaves of plant materials were dried in an oven with internal air circulation (40 °C) (QUIMIS, model Q317M42) for 7 days and pulverized in a rotating knife mill (WILLYE TECNAL, model TE 650) [19]. A weight of 75 g of pulverized plant material were obtained from *Justicia pectoralis* Jacq. The powder thus obtained was duly identified, packaged, and stored at room temperature until used in the experiments. This step was carried out at UniEVANGÉLICA’s Biodiversity Research Laboratory (LAPEBIO). The crude extracts were obtained from the dried and pulverized material of the leaves using the dynamic cold maceration technique [20]. Five extractions with methanol were performed, with an interval of 72 h between them, over a period of 45 days, using the same material (75 g). A volume of 700 mL of solvent was used in each extraction, that is, 0.35 g/mL (*m*/*v*). Then, the extract was concentrated at reduced pressure in a rotary evaporator (45 °C) and the extracts were stored in a freezer (−10 °C) until used in the experiments. A weight of 62 g of crude methanolic extract (78.9%) was obtained.

### 2.3. Animals

A total of 106 adult male Swiss mice (6–8 weeks, 25–35 g) obtained from the colony of the UniEVANGÉLICA’s Central Animal Facility were used in this study. Animals were maintained under temperature-controlled conditions (21 ± 2 °C) with a 12 h light/dark cycle, humidity (60 ± 1%) and free access to water and standard chow. All experiments were carried out between 8:00 a.m. and 4:00 p.m. The experimental protocol was approved by the Ethics in Animal Use Committee (CEUA) of UniEVANGÉLICA (registration n° 002/2020).

### 2.4. Chemicals and Drugs

The following drugs and chemicals were used: indomethacin (Sigma Chemical Company, St. Louis, MO, USA); cimetidine (Teuto, Anapólis, Brazil), Tween^®^ 80 (Sigma-Aldrich), methanol (Merck, PA, USA, EMSURE^®^ ACS ISO Reag. Ph Eur) and CO_2_ (White Martins, São Paulo, SP, Brazil).

MEJP was first solubilized in filtered water with Tween^®^ 80 (2%) at different concentrations (10 mL/kg) of body weight. The control group was treated with vehicle: water filtered with Tween^®^ 80 (2%). Indomethacin was dissolved in sodium bicarbonate solution (5%). All drugs and reagents were prepared immediately before use and dissolved in distilled water.

### 2.5. Indomethacin-Induced Gastric Mucosal Lesions

The animals’ gastric mucosa lesions were induced by indomethacin, according to the method adapted from Djahanguiri (1969) [21]. After a 16 h fast, the animals, the mice were divided into groups (n = 8), vehicle (negative control, filtered water with 2% Tween^®^ 80, p.o. (*per oral*), MEJP at three different dosages (250, 500 and 1000 mg/kg, p.o.) or cimetidine (positive control, 50 mg/kg, p.o.). After 60 min, all groups received indomethacin (50 mg/kg s.c.) dissolved in 5% sodium bicarbonate in distilled water. Six hours after indomethacin administration, the animals were euthanized, and the stomachs were excised and opened along the greater curvature to examine the lesions. The parameters for determining the lesion index were the color of the mucosa, loss of mucosal folds and mucus, edema, petechiae and the number of ulcers, according to Table 1, described by Martins et al. (2014) [1].

### 2.6. Hypothermic Containment Stress Ulcer

The antiulcerogenic activity of MEJP in the gastric ulcer model induced by hypothermic restraint stress was evaluated in mice by the method of Levine (1971) [22] with modifications. After a 16 h fast, the animals (n = 8) received vehicle (2% Tween 80, 10 mL/kg), MEJP (500 mg/kg), or ranitidine (50 mg/kg) by gavage. After 60 min of treatment, gastric ulceration was induced by immobilizing the animals in a closed cylindrical cage maintained at 4 °C and after euthanasia for 2 h, the stomach was removed for evaluation of the lesions index (LI). LI and percent inhibition were calculated. For LI determination, the stomach was opened along the greater curvature and the inner surface was examined with the aid of a microscope. The degree of lesion formation was performed as described by Martins et al. (2014) [1] with few modifications (Table 1).

### 2.7. Pyloric Ligation-Induced Gastric Ulcer Model

The procedure for inducing ulcers by pyloric ligation was an adaptation of the method by Shay et al. (1945) [23]. The mice (n = 8), fasted for 16 h with free access to water containing 5% glucose, were anesthetized with ether and placed in dorsal decubitus on a cork plate. Through an incision of about 2 cm in the abdomen, the stomach was located and the pylorus was ligated with cotton thread. By the intraduodenal route (i.d.), the animals received vehicle (2% Tween^®^ 80, 10 mL/kg), MEJP (500 mg/kg), or ranitidine (50 mg/kg). Then, the abdominal wall was sutured and four hours after surgery, the animals were euthanized in a CO_2_ chamber and their stomachs were removed, washed, and dried in gauze and kept in a beaker with ice-cold saline. After being opened along the lesser curvature, the gastric contents were discarded and the mucous membranes were gently washed with saline. The stomachs were kept in a beaker with ice-cold saline until inspection in a stereoscope. The LI and the number of ulcers were determined as described in item 3.5.1. and results expressed as mean ± mean standard error for each experimental group.

### 2.8. Evaluation of Gastric Acid Secretion Parameters

Groups of mice were treated as described in item 2.7 (Shay et al. (1945) [23]. Four hours later, the animals were euthanized in a CO_2_ chamber, the stomachs were removed, opened along the lesser curvature and the gastric contents were collected, it was centrifuged for 15 min at 2000× *g*, and then the volume (mL), free acidity (pH) and total acidity were quantified.

### 2.9. Determination of Mucus Adhered to the Gastric Mucosa

The modified method of Corne et al. (1974) [24] was used to determine the mucus adhered to the gastric mucosa. After 16 h of fasting, the animals (n = 6) received the vehicle, MEJP (500 mg/kg) or carbenoxolone (200 mg/kg) orally. After 60 min of treatments, the harmful agent ethanol (75% EtOH, 10 mL/kg, v.o.) was administered. After 60 min of administration of 75% EtOH, the animals were euthanized in a CO_2_ chamber and the segments of the glandular region of the stomachs were weighed and transferred to test tubes containing 7 mL of Alcian blue 0.1%. After two consecutive washes with 5 mL of sucrose (0.25 M) and 0.16 M of sucrose in 0.05 M of sodium acetate, (pH 5.8), 5 mL of magnesium chloride (0.5 mol/L) was added to each test tube for extracting the mucus content with the dye. The glandular segment remained in this solution for 2 h, with intermittent agitation. Then, 3 mL of the resulting Alcian blue solution was stirred vigorously with 3 mL of ethyl ether to form an emulsion and centrifuged at 3600× *g* for 10 min to separate the aqueous phase and discard the residue. The Alcian blue concentration in the samples was determined by spectrophotometric reading at 595 nm. The concentration of Alcian blue bound to mucus was determined by interpolation on the Alcian blue standard curve and the results were expressed in μg of Alcian blue/g of tissue.

### 2.10. Cytokine Levels Assay

The gastric mucosal tissue homogenate obtained from the mucus model adhered to the gastric mucosa was centrifuged (10,000 rpm, 4 °C, 5 min) and the cytokines were detected in the supernatant using ELISA kits for TNF-α, IL-6, IL-1β, and IL-10 according to the manufacturer’s instructions at 450nm (R&D Systems, Minneapolis, MN, USA). The plates were read using a microplate reader SpectraMax i3 (Molecular Devices, San José, CA, USA).

### 2.11. Statistical Analysis

To compare the significance level between the two groups, an unpaired Student’s t-test was used as described by Drummond and Tom [25,26]. To compare more than two groups, one-way ANOVA followed by the Tukey post hoc test was used. All analyzes were performed using the GraphPad Prism 9 software (GraphPad Software, La Jolla, CA, USA) for windows. Effects were considered significant for *p* ≤ 0.05.

## 3. Results

### 3.1. Effect of MEJP in Indomethacin-Induced Gastric Mucosal Lesion

The effects of MEJP (250 mg/kg, 500 mg/kg and 1000 mg/kg p.o.) in models of indomethacin-induced gastric ulcer are shown in Table 2. In this model, the administration of MEJP showed significant gastroprotection. In addition, the drug used as a positive control (cimetidine) showed a reduction in the rate of lesions in the induced ulcer model employed.

### 3.2. Effect of MEJP on Stress-Induced Gastric Mucosal Lesion

The results obtained also showed that treatment with MEJP (500 mg/kg, p.o.) or ranitidine (50 mg/kg, p.o.) significantly reduced the IL when compared with the control group, in gastric ulcer induced in the stress model, as shown in Table 3. Based on the percentage of lesion index, the treatment with MEJP or ranitidine reduced it by 31% and 41.5%, respectively.

### 3.3. Effect of MEJP on Gastric Mucosal Lesion Induced by Pyloric Ligation

The model of gastric mucosal lesion induced by pyloric ligation showed that there was a significant reduction in the IL, in the intraduodenal treatment with MEJP or ranitidine, when compared to the vehicle (Table 4).

### 3.4. Effect of MEJP on Assessment of Gastric Secretion Parameters

**Figure 1:**
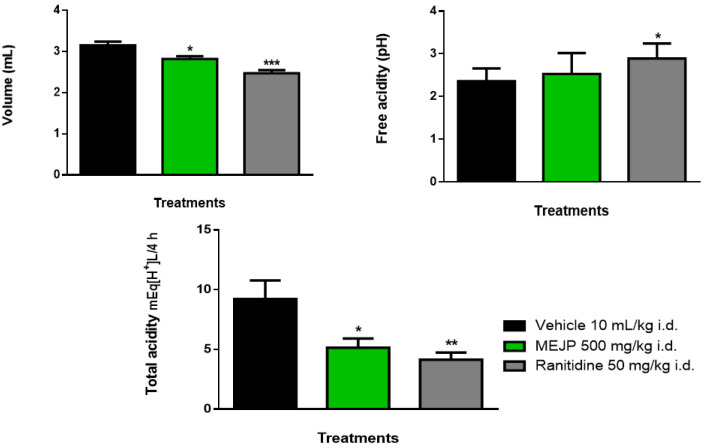
Effect of intraduodenal treatment with MEJP on gastric mucosal lesions induced by pyloric. Values are expressed as mean ± mean standard error (n = 8/group). * *p* ≤ 0.05, ** *p* ≤ 0.01, *** *p* ≤ 0.001, compared to vehicle (control group) using one-way ANOVA and Tukey post hoc test.

Treatment with MEJP was able to decrease the volume of gastric secretion and total acidity, as well as treatment with ranitidine, compared to the vehicle, as shown in Figure 1. When evaluating the free acidity (pH), just ranitidine was able to increase the pH compared to the vehicle (Figure 1).

### 3.5. Effect of MEJP on Determination of Adhered Mucus to Gastric Wall

The Alcian blue binding capacity of gastric mucus in the control group with lesion (ethanol 75%, 10 mL/kg p.o.) was decreased significantly as compared to control group without lesion. However, treatment with MEJP not significantly enhanced the Alcian blue binding capacity of gastric wall mucus. Carbenoxolone (200 mg/kg, p.o.) significantly increased in the binding capacity of Alcian blue mucus from the gastric wall (Table 5).

### 3.6. Effect of MEJP on Cytokine Level Assay

**Figure 2:**
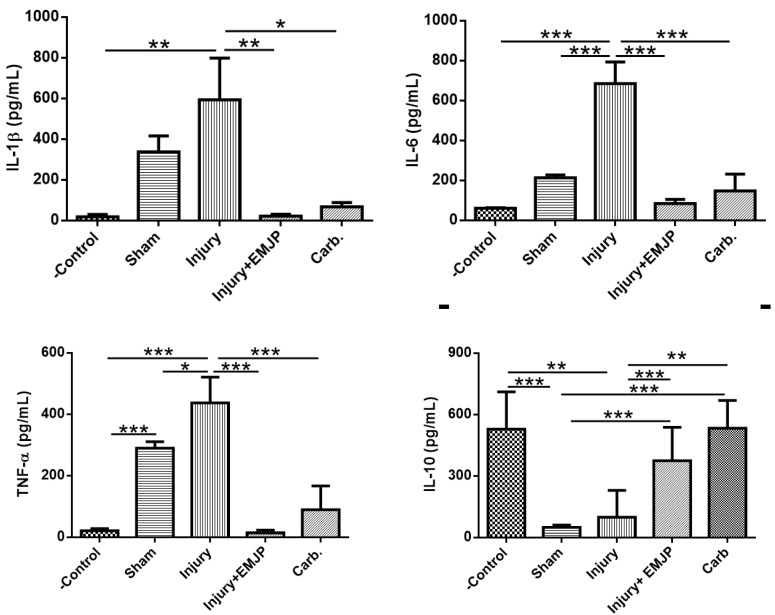
MEJP positively modulates the balance among pro-inflammatory and anti-inflammatory cytokines in gastric tissue. The results are expressed as pg/mf protein and reported as the mean ± standard error (n = 8/group). ANOVA followed by Dunnett’ test. * *p* < 0.05, ** *p* < 0.01, *** *p* < 0.001.

Oral administration of 75% EtOH resulted in the release of large levels of important inflammatory cytokines. MEJP oral administration exerted an effectively downregulatory effect in the gastric mucosa (Figure 2) by reducing the level of inflammatory cytokines IL-1β (reduction about 96.39%, *p* < 0.05), TNF-α (reduction of 92.61%, *p* < 0.05) and IL-6 (a decrease of 89.97% for carbenoxolone, *p* < 0.01) and increasing the level of anti-inflammatory cytokine IL-10 (carbenoxolone enhanced about 78.18%, *p* < 0.001). Thus, data suggest that MEJP inhibited the gene expression of pro-inflammatory cytokines in gastric tissue and thereby mitigated the gastric inflammation.

## 4. Discussion

Although current therapy is effective in the treatment of peptic ulcers, it is notable that the disease still has a high prevalence and continues to be a health problem worldwide. In this way, it becomes necessary to investigate natural medicinal plants and their derivatives as alternatives for therapy and the minimization of side effects [2,27].

There are several mechanisms involved in the pathophysiology of a gastric ulcer; therefore, it is not possible to propose only a single pharmacological mechanism for the antiulcer effects of a given drug [28].

Indomethacin is part of the class of non-selective NSAIDs, which are widely used in inflammatory processes, pain, and fever [29]. However, several adverse reactions are attributed to this pharmacological class, especially peptic ulcers [3,30]. About 90% of ulcer development is attributed to these medications [29]. All NSAIDs cause some degree of gastrointestinal toxicity, although the COX-2 inhibitors diclofenac, associated ibuprofen and naproxen are associated with an increased risk of gastrointestinal lesions [31]. A study carried out by Guzmán-Gómez demonstrated that the administration of indomethacin 40 mg/kg produced accentuated damage in the gastric epithelium of rat stomachs, evidenced by a loss of mucus and the presence of elongated lesions with intense bleeding.

Non-selective NSAIDs inhibit the production of cyclooxygenases, with two isoforms (COX-1 and COX-2), of which COX-1 is called constitutive (responsible for gastric epithelial cytoprotection and hemostasis) and COX-2 is called inducible [3]. The inhibition of COX-1 decreases the level of PGs, consequently reducing the protection of the integrity of the gastric mucosa [3,30,32].

PGE_2_, which is produced from arachidonic acid, is well recognized for having an important protective factor involved in the repair of damaged gastric mucosa, as it exerts a vasodilator effect, as well as increasing blood flow to the surface of the stomach mucosa, in addition to from inhibiting to strengthening gastric acid [33].

The present study investigated the gastroprotective activity of MEJP, in an experimental model of gastric ulcer induced by indomethacin. The result obtained with MEJP indicates the gastroprotective properties of *Justicia pectoralis* Jacq. and its use in the treatment of gastric disorders. As seen in Table 2, doses of 250, 500 and 1000 mg/kg of MEJP demonstrated a reduction in the rate of lesions and an increase in protection compared to the control, although no dose–effect relationship was provided. Therefore, we decided to work with the maximum dose in other tests, as it proved to be a safe dose and suggestive of an effect.

Oxidative stress is involved in the pathogenesis gastric mucosal lesions, which is caused by the imbalance between ROS and antioxidants [34]. It is known that stress can increase the expression of mRNA and pro-inflammatory markers TNF-α, COX-2 and iNOS in the gastric mucosa [34].

The recent study by Di et al. (2020) defines that the high concentration of hydrogen ions is one of the most relevant aggressive factors that facilitate gastric damage, since they decrease the pH in the gastric juice [35]. Treatment of animals with MEJP reduced the volume of gastric secretion as well as the total acidity. Ulcers induced by pyloric ligation are released by self-digestion of the gastric mucosa and in this way favor the impulse of gastric distress in the stomach and decrease protective factors.

TNF-α and IL-1β are pro-inflammatory cytokines responsible for the activation and migration of inflammatory cells to the gastric mucosa, which causes a gastric inflammatory process in addition to being a potent inhibitor of gastric acid secretion. Both cytokines participate in the activation of polymorphonuclear neutrophil leukocytes, in addition to favoring the inflammatory infiltration of lymphocytes and macrophages in the gastric tissue after acute exposure to ethanol [36].

Natural products have been investigated and have shown promising results for their potential gastroprotective effects [37]. Multiple factors are involved in the protective mechanisms of the gastric mucosa [3]. In the present study, MEJP protected the gastric mucosa against the effects caused by COX inhibition caused by indomethacin. This may be related to a specific action by the constituents of the MEJP in the PG pathway.

It is known that an ethanol-induced gastric ulcer is related to local and systemic changes in the production of pro-inflammatory cytokines and that elevated levels of pro-inflammatory cytokines are suggestive of a local inflammatory response evidenced by cellular infiltration [38]. The use of EtOH may intensify gastric mucosal damage via downmodulating the levels of defensive factors, which contribute to the improvement in gastric mucosal blood flow and mucosal microcirculation [39].

Different pro-inflammatory cytokines and enzymes can be used as biomarkers of gastric damage, among which we highlight the TNF-α, IL-1β, IL-6, iNOS and COX-2 genes. Our work showed that after the induction of the inflammatory stimulus induced by EtOH, the inflammatory mediators were significantly increased in the stomach [40].

It is known that the secretion both TNF-α and IL-6 enhances the effects of oxidative stress by inducing mitochondrial ROS generation and cytotoxicity [41]. Previous studies have shown that Zn (II)-curcumin significantly inhibited TNF-α and IL-6 mRNA expression, as well as increasing the activity of SOD and GPx [42]. In this way, the present study demonstrated that MEJP induced the release of IL-10. IL-10 is one of the most important anti-inflammatories and immunosuppressive cytokines, responsible for suppressing the inflammatory response and inhibiting the production of TNF-α.

## 5. Conclusions

The data obtained demonstrate that MEJP was able to reduce inflammatory mediators (TNF-α, IL-1β, IL-6) and increase defensive factors (PGs, IL-10) in gastric tissues after exposure to ethanol, which could attenuate the response inflammation and improve the flow of microcirculation of the gastric mucosa.

Our data also contribute to validate the traditional use the species for gastric disorders and provide a pharmacological basis for the potential clinical; however, other experimental models of induced gastric ulcers are needed, in addition to the isolation of the active compound(s) present in this plant, to elucidate other possible mechanisms of gastroprotection and to determine which phytochemical compounds are involved in this activity.

## Figures and Tables

**Table 1:** Scale by attribution of scores for degree of ulceration.

Lesions	Scores
Discoloration of mucosa	1
Edema	1
Hemorrhages	1
Number of petechia	
Until 25%	2
More than 25%	3
Ulcers or erosion up to 1 mm	*N* × 2
Ulcers or erosion larger than 1 mm	*N* × 3
Perforated ulcers	*N* × 4

*N*: number of stomach lesions.

**Table 2:** Gastroprotective effect of MEJP on indomethacin-induced gastric mucosal lesions.

Treatmant (v.o.)	Lesions Index	Reduction
Vehicle 10 mL/kg	12.88 ± 0.91	-
MEJP 250 mg/kg	7.00 ± 0.42 ***	45.65%
MEJP 500 mg/kg	8.11 ± 0.67 ***	44.80%
MEJP 1000 mg/kg	7.70 ± 0.5 ***	40.22%
Cimetidine 50 mg/kg	6.66 ± 0.44 ***	48.29%

Values were expressed as mean ± mean standard error (n = 9/group). *** *p* ≤ 0.001 compared to vehicle (control group) using one-way ANOVA and Tukey post hoc test.

**Table 3:** Effect of oral treatment with MEJP on stress-induced gastric mucosal lesion.

Treatment (p.o.)	Lesion Index	Reduction
Vehicle 10 mL/kg	10.27 ± 0.64	-
MEJP 500 mg/kg	7.08 ± 0.30 ***	31.06%
Ranitidine 50 mg/kg	6.00 ± 0.32 ***	41.58%

Values were expressed as mean ± mean standard error (n = 8/group). *** *p* ≤ 0.001 compared to vehicle (control group) using one-way ANOVA and Tukey post hoc test.

**Table 4:** Effect of intraduodenal treatment with MEJP on gastric mucosal lesions induced by pyloric ligation.

Treatment (i.d.)	Lesions Index (IL)	Reduction
Vehicle 10 mL/kg	8.07 ± 0.66	-
MEJP 500 mg/kg	6.58 ± 0.35 *	18.46%
Ranitidine 50 mg/kg	6.18 ± 0.26 *	23.42%

Values are expressed as mean ± mean standard error (n = 8/group). * *p* ≤ 0.05 compared to vehicle (control group) using one-way ANOVA and Tukey post hoc test.

**Table 5:** Effects of pre-treatment with MEJP or carbenoxolone on the mucus adhered to the gastric mucosa in mice, in gastric lesions using 75% EtOH.

Groups	Treatment	Dose	Alcian Blue Bound (µg/g Tissue)
Control without lesion	Vehicle + water	10 mL/kg	26.68 ± 2.70
Control with lesion	Vehicle + ethanol	10 mL/kg	11.31 ± 1.13 ***
Carbenoxolone	Carbenoxolone + ethanol	200 mg/kg	18.61 ± 2.10 ^#^
MEJP	MEJP + ethanol	500 mg/kg	12.25 ± 1.48

Values were expressed as mean ± mean standard error (n = 6/group). ^#^ *p* ≤ 0.05 compared to injured control group (CCL) using one-way ANOVA and Tukey post hoc test. *** *p* ≤ 0.001 compared to the uninjured control group (CSL) using one-way ANOVA and Tukey post hoc test.

## Data Availability

Data generated or analyzed during this study are provided in full within the published article and will be available upon a personal request to the corresponding author.
